# Better sleep quality and higher physical activity levels predict lower emotion dysregulation among persons with major depression disorder

**DOI:** 10.1186/s40359-023-01213-3

**Published:** 2023-05-24

**Authors:** Leeba Rezaie, Ebrahim Norouzi, Alexandra J. Bratty, Habibolah Khazaie

**Affiliations:** 1 Sleep Disorders Research Center, Kermanshah University of Medical Sciences (KUMS), Kermanshah, Iran; 2 AB Research Consulting, Las Vegas, United States

**Keywords:** Sleep quality, Major depression disorder, insomnia, Physical activity

## Abstract

**Background:**

People with Major Depression Disorders (MDD) often complain about sleep problems and experience emotion dysregulation. Prior research suggests physical activity can improve both sleep quality and emotional control. However, there is limited research on emotion regulation and the impact of physical activity and sleep in this population.

**Objectives:**

The present study examined the relationships between sleep quality, emotion regulation, and physical activity levels among patients with MDD.

**Methods:**

The sample consisted of 118 patients with MDD (mean age: 31.85 years) who completed questionnaires on sleep quality, physical activity, emotion regulation, and depression.

**Results:**

Results showed that more sleep problems were associated with worse emotion dysregulation, and more physical activity was associated with fewer sleep problems and less emotion dysregulation. Furthermore, physical activity and sleep quality significantly predicted emotion dysregulation, with physical activity being the stronger predictor.

**Conclusions:**

Results from this study suggest that individuals with MDD who are able to engage in physical activity and get better sleep could experience emotional regulation benefits.

## Introduction

Major depression disorder (MDD) is a serious mood disorder which is associated with psychomotor reduction, sadness, hopelessness, diminished interest and physical symptoms, such as chronic pain or digestive issues [1]. People with MDD report a broad range of physical and psychological sleep disturbances [2, 3], or emotion dysregulation [4]. Emotion dysregulation is defined as disruption of both internal and external processes that recognize emotional responses [5].

Sleep disorder can affect overall health and increase inflammatory markers such as cytokines. Higher levels of cytokines lead to increased inflammation in the peripheral and central nervous systems [6], which is often found in people with MDD [7]. Additionally, poor sleep is associated with emotional problems [8, 9] and decreased cognitive control [10], all of which have been reported in this population. Among people with MDD, poor sleep is associated with suicide ideation [11, 12] and cognitive impairments [13]. A review of prior studies suggests that higher physical activity levels are associated with better sleep [14–16]. However, there has been relatively little research on the level and impact of physical activity in people with MDD [17]. Moreover, the relationship between sleep, physical activity, and emotion regulation has not been assessed among people with MDD [18, 19].

Sleep disorders are associated with emotional and cognitive dysfunction [20]. These dysfunctions make it difficult for individuals to disengage from the process of negative thinking, which is the main reason of sleep disorders [21]. Indeed, Norouzi, Rezaie, Bender and Khazaie [22] found that emotion dysregulation factors are significant in sleep disturbances. Moreover, Sadeh, Keinan and Daon [23] found emotional dysregulation, including non-acceptance reactions, predicted a shorter length of sleep. Thus, bidirectionally, sleep problems make it difficult for people with depression to regulate emotions and control cognitions, and this difficulty with emotional and cognitive control further contributes to sleep problems. This downward spiral-like experience is why emotion dysregulation and insomnia severity are crucial targets for understanding and treating depression [22].

Patients with depression often demonstrate a particular style of thinking called rumination, which involves repeated and intense thinking about the causes, consequences, and symptoms of their negative emotions [24]. However and most interestingly, there is evidence that physical activity can reduce the rumination [25], likely by impacting cognition, mood and emotion. For instance, Choi and Salmon (1995) found that regular exercisers have greater mood and emotion stability compared to non-exercisers. Therefore, and based on previous studies, physical activity should be considered as a therapeutic intervention for depressed patients [26, 27].

Previous studies among people with Multiple Sclerosis [28], adolescents [29], and older people [30] found that a sedentary lifestyle was associated with poor sleep quality. However, psychological factors such as emotional dysfunction and depression were not examined. The present study aimed to investigate sleep quality and physical activity levels among people with MDD and the association between sleep quality, physical activity, and emotion regulation. To our knowledge, no research has examined how physical activity and sleep quality might predict emotion dysregulation among patients with MDD.

In the present study, four hypotheses were tested. First, we assumed that lower sleep quality would be positively associated with lower emotion dysregulation. Second, we expected that higher physical activity levels would be correlated with lower emotion dysregulation. Third, we expected that higher physical activity levels would be associated with higher sleep quality (the first three hypotheses were evaluated using correlation analyses). Fourth, we expected that sleep quality and physical activity would predict emotion dysregulation based on a regression analysis.

## Methods

### Participants

The participants were 118 people (mean age: 31.85, *SD* = 7.81 years) with major depressive disorder (diagnosis time: from May 23rd to July 23rd, 2021) who had records at Farabi Hospital (Kermanshah, Iran) and were invited to participate via phone in the present study. Individuals were eligible if they met the following inclusion criteria: (1) diagnosis of a major depressive disorder by a psychiatrist based on the Structured Clinical Interview for DSM-5 [31] and by a self-reported score of ≥ 20 from the Beck Depression Inventory [32], (2) ability to participate in a physical activity intervention, (3) age range between 18 and 80 years, and (4) no history of hospitalization and previous diagnosis of a major depressive disorder. Exclusion criteria included (1) cardiovascular disease, (2) recent head injury, (3) personality disorder, 5) substance use disorder, 6) severe bipolarity with psychotic symptoms, 7) suicidal ideation and intent, and 8) severe sensorimotor injury.

### Procedure

People with MDD were provided information about the purpose of the study, assured that their data would remain confidential, and were asked to review and sign an informed consent form. Participants were asked to complete a series of questionnaires including socio-demographic information, physical activity levels, sleep quality, and emotion dysregulation. The Kermanshah University of Medical Sciences ethics committee approved the study protocol and its amendments (Ethic code: IR.KUMS.REC.1400.153). Additionally, the study adhered to the ethical principles based on the Declaration of Helsinki and its later amendments (2013). The study was conducted from July to September 2021.

## Measures

### Sleep quality

For sleep quality assessment, the Persian version of the Pittsburgh Sleep Quality Index (PSQI) was used [33]. The PSQI consists of 7 items to define sleep quality including (1) subjective sleep quality, (2) latency in sleep, (3) total duration of sleep, (4) habitual sleep efficiency, (5) disturbances in sleep, (6) sleep medication variables and (7) poor daytime functioning. Respondents answer on a 4-point Likert-type scale, ranging from 0 (*very good/no problem at all*) to 3 (*very bad/very big problem*). These seven components are added to create a global score which ranges from 0 to 21. A score higher than 6 indicates poor sleep quality [34]. Previous research validated the Persian PSQI, which had good internal consistency (Cronbach’s alpha = 0.83). The Cronbach’s alpha for the present study sample was also acceptable (α = 0.85).

### Emotion dysregulation

A Persian version of the difficulties in emotion regulation scale (DERS) was used to assess emotion dysregulation [35]. This is a thirty-six item scale measuring levels of difficulty in emotion regulation and cognition across six domains including (1) refusal to accept negative emotions, (2) inability to engage in goal directed behaviors, (3) struggling to manage impulsive behaviors, (4) limited coping strategies, (5) lack of emotional awareness, and (6) lack of emotional clarity. Each item is scored on a 5-point Likert type scale from 1 (*almost never*) to 5 (*almost always*). Emotion dysregulation is measured as a global construct. The higher the DERS’s score, the lower the emotion regulation [36]. The overall internal reliability of the original DERS questionnaire was α = 0.93, and for each subscale it ranged from α = 0.80 to α = 0.89. The internal reliability of the Persian version of DERS in this study was acceptable (α = 0.91).

### Physical activity

A Persian version of the short form of the International Physical Activity Questionnaire (IPAQ-SF) was used to measure physical activity [37]. Participants reported their physical activity levels as either vigorous or moderate. Vigorous physical activity was defined as activities that need maximum physical effort, make breathing hard, cause perspiration, and result in feeling tired. Moderate physical activity was defined as activities that require light physical effort, breathing is normal, and only make people feel a bit tired [38]. Participants were asked to report how often (0–7 days) and for how long (hours) they engaged in vigorous or moderate physical activity before 4 pm. Internal reliability of the Persian version of this scale was satisfactory (α = 0.82).

### Statistical analysis

Descriptive statistics for mean and standard deviations were reviewed. The relationships between sleep quality, physical activity, and emotion dysregulation were examined via a series of Pearson’s correlations and a multiple linear regression test. All statistical analyses were conducted with SPSS version 25 for Lenovo ®. The level of significance was set at alpha < 0.05.

## Results

The descriptive statistics for sample characteristics are highlighted in Table 1. Participants’ mean age was 31.85 years. In addition, two-thirds of participants were female (*n* = 79) and more than half (*n* = 73) were married.

**Table 1:** The mean and standard deviation for the study sample characteristics and variables

Variables	M (SD)
Age (Years)	31.85 (7.81)
BMI (Kg/m2)	26.5
global PSQI score	**11.93 (4.75)**
Total time of sleep	1.92 (0.87)
sleep efficiency	2.54 (1.02)
sleep disturbances	2.2 (0.92)
use of sleeping medication	2.3 (1.01)
subjective sleep quality	0.84 (0.12)
daytime dysfunction	2.1 (0.23)
Emotional Dysregulation	**115.83 (24.59)**
unacceptance emotion response	18.32
Difficulty engaging in Goal-directed behavior	16.43
Impulse control difficulties	19.43
Lack of emotional awareness	23.81
Limited access to coping strategies	22.72
Lack of emotional clarity	15.28
Physical activity levels	**2.11 (1.33)**

Table 2 shows the Pearson’s correlation coefficients between sleep quality dimensions, emotion dysregulation, and physical activity levels, age and body mass index (BMI). Results showed a significant negative association between sleep problems and physical activity levels and emotion dysregulation and physical activity, meaning the greater the physical activity score, the lower the scores for sleep problems and emotion dysregulation. Additionally, there was a significant positive correlation between emotion dysregulation and some of the sleep problem scores, indicating that as sleep problem scores grow, so too does emotion dysregulation, and vice versa. The global sleep quality score was significantly positively associated with global emotional dysregulation, and each of the subscales: unacceptance of emotions, difficulty engaging in goal setting behavior, impulse control difficulties, lack of emotional awareness, and limited access to emotion regulation strategies. There were no significant correlations between sleep problems and age or BMI.

**Table 2:** Pearson’s correlation coefficients between sleep items and age, BMI, Emotional dysregulation, non-acceptance of emotional response, difficulty engaging in goal directed behavior, impulse control difficulties, lack of emotional awareness, limited access to emotion regulation strategies, lack of emotional clarity and self-reported physical activity

variables		Age	BMI	PA levels	Global Emotional Dysregulation score	unacceptance emotion response	Difficulty engaging in Goal-directed behavior	Impulse control difficulties	Lack of emotional awareness	Limited access to emotion regulation strategies	Lack of emotional clarity	
global PSQI score	0.08		0.14	-0.45**	0.49**	0.51**	0.50*	0.52*	0.41*	0.43*	0.31	
sleep efficiency	0.14		0.32	-0.44**	0.21	0.34	0.19	0.33	0.24	0.45*	0.27	
sleep disturbances	0.28		0.21	-0.51**	0.18	0.42*	0.24	0.41	0.40	0.38	0.28	
use of sleeping medication	0.30		0.13	-0.32	0.20	0.12	0.35	0.43	0.24	0.23	0.34	
subjective sleep quality	0.11		0.21	-0.42*	0.44**	0.38	0.45*	0.22	0.34	0.27	0.39	
daytime dysfunction	0.36		0.41	-0.23	0.14	0.23	0.35	0.43*	0.22	0.32	0.30	
PA levels	0.23		-0.37		-0.54**	-0.50*	-0.55*	-0.40*	-0.31	-0.43*	-0.26	

Note: BMI = Body mass index; PA = physical activity; PSQI = Pittsburgh Sleep Quality Index; *= p ≤ .05; **= p ≤ .01

A multiple linear regression was used to predict the criterion variable (emotion dysregulation) from predictor variables (physical activity and sleep problems). The Durbin-Watson statistic (1.89) was in the optimal range (1.5 to 2.5). Therefore, the residuals of the model were uncorrelated or independent. As shown in the Table 3, physical activity and sleep problems significantly predicted emotion dysregulation (*p* < .001). Indeed, the overall regression model, suggested that 62% of the variance in emotion dysregulation could be predicted by physical activity and sleep problems (*R* = .62). Physical activity demonstrated a beta coefficient of -0.421 and sleep problems a beta coefficient of 0.307, suggesting physical activity was a stronger predictor of emotional dysregulation than sleep problems.

**Table 3:** Results of multiple regression test to predict emotion dysregulation through physical activity and PSQI

Predictors	B	β	t	sig
Physical activity	-7.905	-0.421	-5.157	0.001
PSQI	1.619	0.307	3.753	0.001
R = .623, ADJ. R^2^ = 0.378, F(2,115) = 36.509, P < .001, Durbin-Watson = 1.897				

Note = Dependent variable: Emotion dysregulation

## Discussion

The purpose of this study was to examine the relationships between sleep quality, physical activity, and emotion dysregulation among people with MDD. The study findings supported the hypotheses. The first hypothesis assumed a relationship between sleep quality and emotion regulation dimensions, and our data supported this assumption, demonstrating there was a significant positive correlation between the two variables. Thus, if sleep quality is low, so too emotion dysregulation will be low, and vice versa. These data are consistent with previous findings on the relationship between sleep and emotion regulation [21, 39, 40]. This finding can also be interpreted with the cognitive reappraisal and higher-level cognitive functions model [41]. It is well documented that lower sleep quality can disrupt prefrontal functions such as cognitive control, which is very important in emotion regulation. Therefore, sleep would be an important factor for emotion regulation [40]. Indeed, dysfunctional emotional-cognitive processes such as limited coping strategies and a lack of emotional recognition that can obstruct self-regulatory behaviors are a contributing factor to insomnia [42].

Sleep plays a critical role in psychosocial and emotional adjustment. There is evidence that poor sleep can decrease emotion-related processes and several aspects of neurocognitive functioning. For example, neurobiological theory posits that subcortical brain structures of the limbic system, the Insula and Amygdala, generate emotional responses to stimuli controlled by the prefrontal cortex. Therefore, emotion and behavior are regulated by both prefrontal cortex and the Amygdala [43, 44]. Consequently, it seems that emotion dysfunction may co-occur with sleep abnormalities [43].

Regarding the second hypothesis, we expected that higher physical activity levels would be significantly correlated with lower emotion dysregulation scores, and the data supported this assumption. Moreover, this finding was aligned with previous studies that demonstrated a similar connection [45, 46], and other research demonstrating a general positive association between physical activity and emotional stability [47, 48]. There is also evidence that physical activity can change the way a person responds to emotional events. Based on physiology theory, physical activity may help individuals return to pre-stress levels more quickly [48]. Therefore, when people are active they can cope with emotional stress effectively. Furthermore, greater physical activity facilitated the down-regulation of negative emotions and mood symptoms among people with emotional distress [49]. This claim is consistent with our study that showed higher physical activity is associated with lower emotion dysregulation.

The third hypothesis expected that higher physical activity levels would be associated with better sleep quality, and these data supported the assumption. The present result was consistent with other cross-sectional studies showing physical activity is associated with more favorable sleep quality [50, 51]. Additionally, the current findings are in line with studies on healthy adolescents [29] and clinical samples [52, 53]. An active lifestyle is one component that can help reduce rumination [54], which is one of the key factors in sleep disorders [55]. The energy expenditure theory helps explain the relationship between physical activity and sleep [56]. Energy expenditure is hypothesized that physical activity can increase energy expenditure in wakefulness to regulate total energy needs during sleep. Moreover, physical activity can improve sleep quality by increasing neurobiological factors, and regulating body temperature, which is an important factor in sleep [57].

Finally, our fourth hypothesis was supported by data showing a significant regression model with 62% of the variance in emotion dysregulation predicted by physical activity and sleep problems in this sample. Overall, these findings support the suggestion that emotional regulation issues may play a role in the mechanism by which sleep disorders impact depression [58].

Of course, there are limitations to these results. First, the sample size was small. Second, we did not assess physiological (serotonin, noradrenaline and dopamine) and psychological factors (e.g. self-determined motivation and stress), or MDD symptoms (depression, anxiety, hopelessness, psychiatry medication, quality of life), which might have influenced outcomes. Third, the cross-sectional design does not allow for any causal understanding of the relationship between the variables examined. Consequently, the present study contributes to the literature but has shortcomings given the purely correlational work. Future research should consider an experimental research design that facilitates cause-and-effect findings, evaluation of depression as a primary symptom of MDD, and inclusion of objective measurements in addition to self-reported factors.

## Conclusions

Among a sample of people with MDD, there were significant correlations between sleep quality, physical activity, and emotional dysregulation. More sleep problems were associated with worse emotion dysregulation, and more physical activity was associated with fewer sleep problems and less emotion dysregulation. Furthermore, physical activity and sleep quality significantly predicted emotion dysregulation, with physical activity being the stronger predictor. Consequently, individuals with MDD who are able to engage in physical activity and get better sleep could experience emotional regulation benefits. The clinical ramifications of these findings are worthy of consideration. Our findings imply that interventions to enhance sleep and/or address maladaptive emotion regulation could help to avert the onset of depressive symptoms.

## Data Availability

The datasets analyzed during the current study are not publicly available due to the fact that data are identified but are available from the corresponding author on reasonable request.
